# The role of basket trials in drug development for neurodegenerative disorders

**DOI:** 10.1186/s13195-022-01015-6

**Published:** 2022-05-25

**Authors:** Jeffrey Cummings, Arturo Montes, Sana Kamboj, Jorge Fonseca Cacho

**Affiliations:** 1 Chambers-Grundy Center for Transformative Neuroscience, Pam Quirk Brain Health and Biomarker Laboratory, Department of Brain Health, School of Integrated Health Sciences, University of Nevada Las Vegas (UNLV), Las Vegas, NV USA; 2 Henderson, NV 89052 USA; 3 Kirk Kerkorian School of Medicine, University of Nevada Las Vegas (UNLV), Las Vegas, NV USA; 4 Department of Neurosurgery, John Hopkins University School of Medicine, Baltimore, MD USA; 5 Department of Computer Science, Howard Hughes School of Engineering, University of Nevada Las Vegas (UNLV), Las Vegas, NV USA

**Keywords:** Neurodegenerative disease, Basket trial, Alzheimer’s disease, Parkinson’s disease, Dementia with Lewy bodies, Multiple system atrophy, Amyotrophic lateral sclerosis, Frontotemporal dementia, Progressive supranuclear palsy, Corticobasal degeneration, Clinical trials

## Abstract

**Background:**

Drug development for neurodegenerative disorders (NDDs) is a long, complex, and expensive enterprise. Methods to optimize drug development for NDDs are needed. Basket trials have been widely used in oncology and have been promoted by the Food and Drug Administration as a means of enhancing the efficiency of drug development.

**Discussion:**

We reviewed clinical trials for NDDs registered on clinicaltrials.gov in the past 10 years. We identified 59 basket trials assessing the impact of treatment on more than one NDD in the trial. Forty-one of the trials were for 25 agents addressing symptoms of NDD such as motor impairment, hypotension, or psychosis. Eighteen of the trials assessed 14 disease-modifying therapies; the principal targets were mitochondrial function, tau biology, or alpha-synuclein aggregation. Basket trials are most common in phase 2 but have been conducted in phase 1, phase 3, and phase 4. The duration and size of the basket trials are highly variable depending on their developmental phase and the intent of the trial. Parkinson’s disease was the most common disorder included in basket trials of symptomatic agents, and Alzheimer’s disease was the most common disorder included in basket trials of disease-modifying therapies. Most of the basket trials of symptomatic agents were sponsored by pharmaceutical companies (29 of 41 trials); similarly, most of the basket trials investigating DMTs in basket trials were sponsored by the biopharmaceutical industry (11/17 trials).

**Conclusions:**

Basket trials may increase drug development efficiency by reducing redundancy in trial implementation, enhancing recruitment, sharing placebo groups, and using biomarkers relevant to the mechanism of action of the treatment across NDDs. There have been relatively few basket trials including multiple NDDs in the same trial conducted over the past 10 years. The use of the basket trial strategy may represent an opportunity to increase the efficiency of development programs for agents to treat NDDs.

## Background

Improvements in healthcare and standards of living have increased the lifespan, with the population of people 60 and over projected to more than double by 2050 [1]. A larger elderly population results in an increased prevalence of neurodegenerative diseases (NDDs) [2]. NDDs comprise a broad category of disorders characterized by nervous system dysfunction, stemming from the death or loss of function of neurons [3]. Of the NDDs, Alzheimer’s disease (AD) is projected to affect up to 81 million people by 2040 [4]. In addition to reducing the quality of life and longevity for patients, NDDs place significant strain on both care partners and healthcare systems. The global cost of dementia in 2015 was estimated at 818 billion dollars [5]. Given the personal impact and resource requirements for care, it is crucial that effective therapeutics be found to alleviate the burden posed by NDDs.

Drug development for NDDs is costly and time consuming. The creation of a new drug to treat an NDD can cost up to $5.7 billion and take up to 13 years [6]. The rate of success in NDD drug development is low; 80% of drug clinical trials fail in phase I or II, and only 1 in 5000 new drugs typically receives approval by the US Food and Drug Administration (FDA) [7]. Only two disease-modifying therapies (DMTs) have been approved for adult NDDs — edaravone for amyotrophic lateral sclerosis (ALS) and aducanumab for (AD) [8, 9] — and a limited repertoire of symptom-relieving therapies are available for patients with NDDs such as cholinesterase inhibitors for AD and dopaminergic agents for Parkinson’s disease (PD). New approaches are needed to accelerate NDD drug development. One such means capitalizes on drugs that are being assessed for treatment of one NDD, address aspects of biology or symptoms shared with other NDDs, and might be evaluated simultaneously across multiple disease states. A drug that is developed for a single NDD may have the potential to treat others. NDDs occur primarily in older individuals and learnings from treating one NDD regarding dose, tolerability, pharmacokinetics, and safety will likely apply across multiple NDDs.

One approach to studying the effects of a single agent across multiple NDDs is to employ “basket” trials. The basket trial design simultaneously evaluates the effects of a targeted therapy on multiple conditions that have a common biology or shared clinical features [10]. Basket trials are designed to assess a single investigational drug or drug combination in different disease populations defined by disease stage, histology, number of prior therapies, genetic or other biomarkers, or demographic characteristics [11]. Basket trials are often used in oncology where several tumors that share a rare mutation or other molecular features are tested in a single trial of an anti-cancer therapy [12]. This strategy has been embraced by the FDA as a means of providing exploratory data that can inform further studies, support marketing applications, or inform the likelihood of success of advancing an agent in a specific disease population [10, 13].

The use of basket trials is less common in neuroscience therapeutic areas than in oncology. We explored the use of basket trials in NDDs represented on clinicaltrials.gov. We describe drugs in clinical trials for multiple NDDs. Drugs in this category were determined through an analysis of all clinical drug trials conducted for 11 prespecified NDDs and updated in the past 10 years as registered on clnicaltrials.gov. We review basket trials for both symptomatic agents such as anti-parkinsonian agents tested in trials including several disorders that manifest parkinsonism and putative disease-modifying therapies (DMTs) that address a common underlying biology across NDDs. We developed automated search and classification algorithms to derive data from clinicaltrials.gov.

### Research methods

Clinicaltrials.gov [14] is a comprehensive registry of clinical trials supervised by the US National Library of Medicine. The registry was established by legislation in 1997 and requires information on the purpose of the experimental agent in the trial, subject eligibility criteria, location of clinical sites conducting the study, and a point of contact for individuals interested in the trial. The legislation was updated in 2007 to require posting of results of the trials within 1 year of completion, and the Final Rule for Clinical Trials Registration was implemented in 2017 to clarify and expand the requirements for clinical trial registration and submission of results [15].

Clinicaltrials.gov allows all study record content to be downloaded for analysis as a zip file. This zip contains an XML file and is structured according to a specified schema. To acquire the information needed for this study, we downloaded the latest version of this file on 06/06/2021 and entered it into a relational database using the same prespecified schema. Next, we performed queries to extract relevant columns in the dataset such as registration number (National Clinical Trial Identification [NCTID]), condition name, intervention name, etc. The results were Comma Separated Values (CSV) files where each line represents one clinical trial, or study, with a unique NCTID. We used Python Programming Language to write scripts to further process the data. The exact queries, along with a copy of all the project code, can be found in our GitHub Repository [16, 17]. The first script, *nddfilter.py*, removes all studies that do not list an NDD under the condition column. We defined NDD studies as those with a condition listed as Lewy body dementia (LBD)/dementia with Lewy bodies (DLB), AD, PD, PD dementia (PDD), frontotemporal lobar degeneration (FTLD) (including frontotemporal dementia (FTD), progressive supranuclear palsy (PSP), and corticobasal degeneration (CBD)), chronic traumatic encephalopathy (CTE), Huntington’s disease (HD), amyotrophic lateral sclerosis (ALS), and multiple system atrophy (MSA). We sanitized the condition name as some studies had spelling variations of these NDD names, misspellings, and alternate name versions. We limited our search to trials with a posted update between 01/01/2010 and 06/06/2021. In addition to identifying NDD in the “condition” area of the trial description, we searched for trials with an NDD listed in the eligibility or inclusion criteria for the trial. With this approach, we were able to detect additional trials of interest that did not identify all NDDs of the trial in the “condition” section of clinicaltrials.gov. The code for processing the data and generating the appropriate tables is in the *nddfilter.py* and in the *eligcritprocesser.py* script.

Next, we created a script, *drugclassifier.py*, that conducts searches and matching for single trials that list two, or more, NDD (condition and/or inclusion criteria) using the same intervention. When these matches were complete, we created a script, *csvClassifier.py*, to remove all trials that do not contain a drug as the intervention type (e.g., devices, behavioral therapies, etc.). We advanced our classifier by training it with data with different intervention types such as drugs, behavioral treatments, biomarkers, devices, stem cells, and supplements, and removal of irrelevant interventions such as “placebo” or false positives from our inclusion criteria search caused by complex wording in the text. Within the drug class, we created sub-classes for symptomatic agents and DMTs. These training data were in the form of carefully curated text files containing samples for each class or sub-class. The resulting entries generated the final tables using our *tablegeneration.py* script. When the strategies were complete, classification required less than 2 s to run on a personal computer. The initial NDD trial extraction from all clinicaltrials.gov trials required less than 5 min. We did not include studies comprised only of AD and mild cognitive impairment (MCI) in the same study, and we did not include trials comprised only of PD, PDD, or PD MCI in the same study. These studies are of similar disorders at different stages of severity and did not meet our definition of basket trials. Similarly, although trial populations labeled as “dementia” may include multiple NDDs, the diagnoses are not specified, and we did not include them in this interrogation for basket trials.

We included all interventional studies including phase 1, phase 2, phase 3, and phase 4 clinical trials that were not yet recruiting, recruiting, enrolling by invitation, active, not recruiting, terminated, completed, suspended, withdrawn, and unknown. We captured information on the trial agent; trial title; trial number in clinicaltrials.gov; start date; projected end date; actual end date; number of subjects planned for enrollment; number of arms of the study; whether a biomarker was described; subject characteristics (age range; acceptable range of cognitive impairment, etc.); and sponsorship (a biopharmaceutical company, National Institutes of Health (NIH) with academic medical centers, public-private partnership, or “other”).

For therapeutic targets, we classified agents using the Common Alzheimer’s Disease Research Ontology (CADRO) classification [18]. Although focused on AD, the CADRO includes basic mechanisms common to all NDDs. CADRO categories include amyloid beta; tau; apolipoprotein E (ApoE), lipids and lipoprotein receptors; neurotransmitter receptors; neurogenesis; inflammation; oxidative stress; cell death; proteostasis/proteinopathies; metabolism and bioenergetics; vasculature; growth factors and hormones; synaptic plasticity/neuroprotection; gut-brain axis; circadian rhythm; environmental factors; epigenetic regulators; multi-target; unknown target; and others. Non-amyloid protein aggregations found in non-AD NDDs were listed under “proteostasis/proteinopathies” if the agent targeted these pathways.

The mechanism of action (MOA) of the agent was derived from the trial description or related literature. Some agents have more than one MOA, and we use the available literature to identify a dominant mechanism. We use the terminology of “symptomatic” treatments for agents whose purpose was motor improvement, control of hypotension, cognitive enhancement, or improvement of neuropsychiatric symptoms without claiming to impact the biological causes of cell death; we used the terminology of “disease-modifying” for treatments that intended to change the biology of the NDD and produce neuroprotection directly or through intermediate mechanisms such as effects on amyloid, tau, or alpha-synuclein [19].

## Results

We identified 59 basket trials assessing 39 drugs that were updated over the past 10 years. Table 1 summarizes the symptomatic agents used in basket trials. The test agent, indications, composition of NDDs in the trials, and the number of trials with each agent are shown.

**Table 1:** Characteristics of basket trials for symptomatic agents in neurodegenerative disorders

***Drug***	***Number of trials***	***Goal of treatment***	***NDDs included***
Alprazolam	1	Reduced anxiety	HD, AD
Ampreloxetine	1	Control of orthostatic hypotension	PD, MSA
Apomorphine	1	Pain control	CBD, PSP
Armodafinil	1	Improved attention, cognitive enhancement	PD, DLB
AVP-786	1	Reduced disinhibition	DLB, AD, PSP, HD, FTLD
AVP-923	2	Control of pseudobulbar affect	AD, PD, HD, ALS
Botulinum toxin	1	Reduction of rigidity	AD, FTLD
Cannabis	2	Improved sense of well-being, improved quality of life; reduction of pain, nausea, vomiting	PD, CTE, ALS
Carbidopa	1	Improved motor function	MSA, PD
Droxidopa	7	Improved motor and non-motor symptoms, increased blood pressure	PD, MSA, PSP
Entacapone	1	Improved motor function	MSA, PD
Incobotulinum Toxin A	2	Reduced salivary volume	PD, ALS, MSA, PSP
Intepirdine (RVT-101)	1	Improved gait	DLB, AD, PD
Lithium	1	Improved quality of life, reduced depression	CBD, PSP
LY31544207	1	Cognitive enhancement	DLB, PD
Memantine	2	Cognitive enhancement	PD, DLB, FTLD, ALS
Midodrine	4	Control of orthostatic hypotension	MSA, PD
MP-101	1	Reduction of dementia-related psychosis	AD, FTLD, PD, DLB
Nebivolol	1	Control of hypotension	MSA, PD
Nelotanserin	3	Reduced REM sleep behavior disorders, reduced visual hallucinations	PD, DLB
NYX-458	1	Cognitive enhancement	PD, DLB
Pimavanserin	2	Reduction of dementia-related psychosis with delusions and hallucinations	AD, PD, DLB, FTLD
Ramalteon	1	Improved sleep efficiency	HD, DLB, PD
Rimabotulinum toxin B	1	Reduced sialorrhea	PD, ALS
TD-9855	1	Reduced orthostatic hypotension	MSA, PD

All the symptomatic agents addressed the CADRO category of transmitter or transmitter receptor function. Droxidopa was the drug most used in symptomatic trials of multiple NDDs; studies in 7 basket trials included PD, PSP, and MSA. Also common in basket trials of symptomatic agents were midodrine (4 trials) for the treatment of hypotension and nelotanserin (3 trials) for the treatment of psychosis. Motor abnormalities of parkinsonism were the most common symptoms studied in multi-NDD basket trials (9 trials); orthostatic hypotension was a common cross-NDD condition assessed (7 trials). PD was the most common disease included in multi-NDD trials of symptomatic agents (targeted by 20 agents); followed by DLB (targeted by 10 agents), MSA (8 agents), AD (7 agents), PSP, ALS, FTD (5 agents each), HD (4 agents), CBD (2 agents), and CTE (1 agent). Trials including PD plus DLB (12 trials) and PD plus MSA (11 trials) were the most common combinations. Four symptomatic agents were in phase 1, 17 in phase 2, 8 in phase 3, and 5 in phase 4 (some agents did not have an assigned phase).

Basket trials of symptomatic agents varied greatly in terms of trial exposure duration with some such as midodrine (NCT02897963) testing the participant within 10 min of change in position on a tilt table and alprazolam (NCT00975481) treatment effects being observed within 30 min of administration and continuing for 24 h. A cannabis trial (NCT03944447) had exposure of up to 5 years. Excluding the trials at the extremes of duration, basket trials of symptomatic agents had an average exposure period of 11 weeks (21 trials). The trial of pimavanserin continued until an interim analysis demonstrated less relapse in the active treatment group compared to placebo in a randomized discontinuation trial involving dementia-related psychosis and including AD, PD, DLB, FTD, and vascular dementia (NCT03325556).

Similarly, the trial size of symptomatic agents assessed in multiple NDDs varied depending on the type of intervention being assessed. A cannabis trial (NCT03944447) collected data using an on-line registry from participants with a wide variety of conditions in addition to NDDs and anticipated a sample size of 200,000 over 5 years; the number of participants with NDD expected in the trial was not specified. A trial of AVP-923 (NCT00056524) focused on the treatment of pseudobulbar affect in AD and PD and included 600 participants in a double-blind parallel-group design. Memantine is approved for the treatment of cognitive impairment in AD and was studied in two trials with PD and DLB (NCT00630500; NCT00855686) for its potential cognitive-enhancing properties in trials with a planned sample size of 75 and 199 participants, respectively. The trial of pimavanserin (NCT03325556) was a phase 3 trial including 392 participants. Excluding the extreme trials, the mean sample size was 120 participants (35 trials).

The biopharmaceutical industry sponsored 29 of the 41 basket trials assessing symptomatic therapies; 12 were sponsored by academic medical centers funded by the NIH.

Eighteen trials assessed 14 drugs for their disease-modifying effect in basket trials involving more than one NDD. Table 2 provides information on the test agent, indications, CADRO target classification, composition of NDDs in the trials, and the number of trials with each agent.

**Table 2:** Characteristics of basket trials for disease-modifying agents in neurodegenerative disorders

***Drug***	***Number of trials***	***CADRO target***	***NDDs included***
ATH-1017	1	Neuroprotection	PD, DLB
BIIB092	1	Tau extracellular transmission	FTLD, CBD, CTE
Davunetide (AL-108, NAP)	1	Tau aggregation	PSP, CBD, FTLD
Deferiprone	1	Oxidative stress	ALS, PD
Insulin	1	Insulin resistance	PD, MSA
Latrepirdine (dimebon)	5	Bioenergetic mitochondrial agent	HD, AD
LMTM (TRx0237, LMT-X)	1	Tau aggregation	FTLD, AD
Metformin	1	Insulin resistance	FTLD, ALS
NBMI (N,N′-bis(2-mercaptoethyl) isophthalamide)	1	Mercury chelation (oxidative stress)	PSP, MSA
Posiphen	1	Protein aggregation	PD, AD
TPI-287 (abeotaxane)	1	Tau microtubule stabilizer	CBD, PSP
Traneurocin (N-831)	1	Neurogenesis	DLB, AD
Warfarin	1	Vascular (anticoagulant)	AD, HD
Zoledronic acid	1	Bone loss prevention	PD, DLB, PSP, MSA

Latrepirdine (dimebon) is an agent with putative DMT properties studied in 5 NDD basket trials. Trials including both AD and HD were conducted. Latrepirdine’s therapeutic target involved mitochondrial activity resulting in the emergence of mitochondrial function and bioenergetics as the most common mechanisms studied in DMT trials of multiple NDDs. Five drugs targeted synucleinopathies (PD, DLB, MSA). Tau was a common CADRO target with 4 trials addressing various aspects of tau biology and one trial (NCT04524351) assessing effects on multiple proteins including tau. Insulin resistance and oxidation were studied in 2 trials each; vascular factors and neurogenesis were studied in 1 trial each. Across all trials, AD and PD were the most common disorders assessed (each targeted by 5 agents); followed by PSP and FTLD (4 agents each); DLB, CBD, and MSA (3 agents each); ALS and HD (2 agents each); and CTE was the target of treatment in 1 trial.

Nine of the DMTs were in phase 1, 6 in phase 2, and 1 in phase 3 (0 in phase 4), with 1 listed as “not applicable.”

Basket trials were of variable length depending on their phase and the intention of the trial. DMTs in phase 1 assessing pharmacokinetics of the test agents were brief, usually 14 days or less. In contrast, the trial of LMTM (NCT02245568) assessing its impact on the treatment of tau biology in AD and FTLD was 34 months in duration. Trials of intermediate duration (7 trials) had a mean duration of 18 weeks (range 4–52 weeks).

Trial size varied from 3500 participants in the trial of zoledronic acid (NCT03924414) and 939 in the trial of LMTM (NCT02245568) to 14–20 in phase 1 pharmacokinetic trials. Excluding the trial not limited to NDDs, the mean size of basket trials of DMTs was 96 participants (16 trials).

Basket trials of DMTs were funded by NIH-academic collaborations (7 trials) or by biopharmaceutical companies (11 trials).

There has been an increase in the use of basket trials with 26 of 41 (63%) basket trials of symptomatic agents and 12 of 18 (66%) basket trials of DMTs conducted in the last 5 years.

Biomarkers play an increasingly important role in drug development and have been included in some basket trials. A study of deferiprone (NCT02880033), an iron-chelating agent, included measures of hydroxyl radical formation, adenosine triphosphate production, oxygen consumption, free reactive iron concentration, and lipid peroxidation. A trial of TPI-287 (NCT02133846) targeting tau-related tubulin-binding and microtubule-stabilization assessed cerebrospinal fluid levels of biomarkers of neurodegeneration including neurofilament light chain, total tau, tau isoforms, and tau phosphopeptides. Magnetic resonance imaging was collected to explore the effects of changes in brain network function, structural connectivity, and perfusion. Posiphen blocks translation of mRNA of both amyloid precursor protein and alpha-synuclein, actions relevant to AD and PD, respectively. Biomarker outcomes are being used in phase 1 of a trial to guide dosing decisions in phase 2 (NCT04524351).

## Discussion

Basket trials are defined by the FDA [13] as a type of master protocol designed to test a single investigational drug or drug combination in different populations defined by disease stage, histology, number of prior therapies, genetic or other biomarkers, or demographic characteristics. While designed primarily for the assessment of cancer therapies, basket trials can allow efficient assessment of drugs in other conditions, including NDDs. The FDA guidance notes that each subpopulation of a basket trial comprises a substudy. Each disease-defined substudy should include specific objectives, the scientific rationale for inclusion of each population, and a detailed statistical analysis plan that includes sample size justification and stopping rules for futility. The FDA encourages the use of master protocols such as basket trials. They note that these protocols can be flexible and incorporate efficiency-oriented approaches such as a shared placebo control arm and centralized data capture. Basket trials can accelerate the recruitment of participants to trials since patients with several types of NDD being seen in clinics can be referred. Basket trials avoid the cost and operational challenges of implementing separate trials for each NDD. The use of basket trials in cancer drug development programs has grown rapidly; their use has been primarily in phase 1b/2 [11]. NDD basket trials have been employed in all development phases with their most common application in phase 2 (46% of NDD basket trials).

There are few programs utilizing basket trials that led to an approved therapy. Droxidopa is approved for the treatment of orthostatic hypotension occurring in multiple NDDs included in the same trials including PD, MSA, and pure autonomic failure [20]. Midodrine is approved for orthostatic hypotension of unspecified etiology not based on basket trials with specific NDD populations. Midodrine is used for the treatment of orthostatic hypotension in NDDs including PD [21].

AVP-923 (dextromethorphan plus quinidine; Nuedexta™) is approved for the treatment of pseudobulbar affect based on individual studies in multiple sclerosis [22] and ALS [23] and well as a basket trial with both disorders [24]. This approach may have allowed the development program to avoid conducting a second trial in each individual disorder typically required for treatment approval. The strategy would decrease cost and shorten the time to FDA approval and marketing. The trials of AVP-923 leading to regulatory approval were conducted prior to the 10-year retrospective analysis reported here. The review identified one trial of AVP-923 for pseudobulbar affect in a basket trial including AD and PD (NCT00056524). The deuterated form of AVP-923 — AVP-786 — was applied in a basket trial investigating the effects on disinhibition and comprised of participants with DLB, AD, PSP, HD, and FTLD.

Rivastigmine is the only agent approved for treatment of cognitive impairment in 2 NDDs — AD and PD dementia [25, 26]. This approval was not the result of a basket trial; separate trials were conducted for each NDD. Identical trial outcomes — the Alzheimer’s Disease Assessment Scale – cognitive subscale (ADAS-cog) and the Alzheimer’s Disease Cooperative Study Activities of Daily Living scale — were used to assess cognition and function, respectively, in both conditions.

The basket trials of symptomatic agents most conducted included dopaminergic agents targeting symptoms of parkinsonism. The trials included carbidopa (1 trial), droxidopa (7 trials), and entacapone (1 trial). One trial (NCT00547911) included carbidopa, droxidopa, and entacapone in the same basket trial.

Disease mechanisms explored through treatment with DMTs included mitochondrial dysfunction in the basket trials of latrepirdine, protein aggregation in trials of synucleinopathies, and tauopathy with agents targeting aspects of tau biology. The relatively small number of each disease-related population of patients with tauopathy may suggest that basket trials are an important design option for efficient drug assessment.

There are several methodologic issues unique to basket trials. If each condition is approached as a substudy within the basket trial, the assessment instruments can be individualized for each disorder. If common measures are desired to attempt a comparison across NDDs, challenges may arise. NDDs have different cognitive profiles, and the use of a single neuropsychological outcome such as the ADAS-cog in basket trials of cognitive-enhancing agents may not capture the cognitive response of different NDDs. AD patients have amnestic type memory deficits, whereas FTD patients have a predominance to executive dysfunction [27]. The differences in cognitive profiles may make it difficult to match patients with different NDDs for severity of clinical deficits that may be required by the trial design. Global measures such as the Clinical Dementia Rating (CDR), CDR-FTLD, or clinical global impression (CGI) [28, 29] may be better suited to capture treatment responses across NDDs [20]. Basket trials of symptomatic agents for the treatment of orthostatic hypotension or parkinsonism may have more uniform responses across NDDs.

Basket trials usually include participant populations that have different background medications that could affect the response to treatment. ALS patients are likely to be on riluzole, and HD patients may be on tetrabenazine which could differentially affect treatment responses in a basket trial [30]. Stability of dosing prior to trial entry and planned analyses of patients on and off these agents warrant consideration in basket trial designs. This issue will be addressed in each condition evaluated as part of a substudy within the basket trial.

Different NDDs may have different safety and tolerability profiles that must be monitored during basket trials. Older patients with AD may have adverse event profiles that differ from those of younger patients with ALS or HD. For example, TPI-287 is a putative microtubule stabilizer that could have efficacy in multiple tauopathies as well as in the treatment of the tauopathy of AD. A basket trial for this agent included PSP, CBD, and AD. AD patients had a disproportionate number of anaphylactoid reactions suggesting that further development of this agent for AD could be problematic [31].

Basket trials may suffer from small sample sizes and limited power as well as different numbers of each NDD in the trial. This may result in limited ability to draw conclusions on the consistent effectiveness of a therapy across NDDs. A trial of pimavanserin (NCT03325556) for the treatment of dementia-related psychosis included five types of dementia with delusions and hallucinations. The trial had a randomized discontinuation design and was powered on the response (e.g., failure to relapse in the treatment group compared to the placebo group) observed in the entire trial population. An adequate number of patients with AD and PD entered the trial to allow subgroup assessments; the small number of patients with vascular dementia, DLB, and FTD make it difficult to draw conclusions concerning these individual sub-populations [32]. Flexible Bayesian analysis of subgroups has been proposed as one means of optimizing data interrogation from subgroups while avoiding spurious conclusions [33].

Bayesian approaches have also been proposed that optimize the ability to terminate a trial for lack of efficacy or for compelling efficacy [34]. Basket trials have been most widely applied in oncology where molecular characterization of subtypes and rapid progression may facilitate the use of basket designs and Bayesian statistics. Adjustments for the characteristics of NDDs — slow progression, older patients, mixed pathology, heterogeneous clinical progression, variable response to treatment — are necessary when planning basket trials in the neurodegenerative therapeutic area.

Biomarkers can play critical roles in basket trials. They can confirm diagnoses such as the use of amyloid imaging to confirm the diagnosis of AD or the use of genetic studies to confirm the diagnosis of HD. Biomarkers can comprise the primary outcome measure of basket trials. Posiphen, an inhibitor of mRNA translation for several proteins, has been shown to reduce alpha-synuclein, tau, and amyloid protein levels in animal models of PD and AD [35, 36]. Posiphen is in a basket trial including patients with AD and PD where biomarker information from part 1 of the trial will guide decisions for part 2 (NCT04524351).

Basket trials can provide important decision support for a development program and can improve efficiency in determining if a candidate treatment can be developed for multiple indications or if one NDD has a more promising efficacy and safety profile than others. Basket trials have their greatest utility in early stages of development programs when these decisions are being made. The predominance of the use of basket trials by biopharmaceutical sponsors shown in this review suggests that basket trials may be most useful in industry-based development programs. They can be considered by academic and governmental sponsors when funding allows sufficient sample sizes to power conclusions for each NDD included in the trial.

This study has limitations. The data on basket trials were derived from clinicaltrials.gov; this database is comprehensive but not exhaustive and some basket trials, especially those performed outside the USA may not have been included. The data available on clinicaltrials.gov may be incomplete and prevented us from including all trials in all analyses (e.g., biomarker data are sometimes lacking). We have not explored the outcomes of basket trials in NDDs. Compliance with the directive to report results within 1 year of trial completion is limited, compromising the ability to draw conclusions from basket trials in clinicaltrials.gov [37, 38]. Additional insights into the efficiency of basket trials may be forthcoming when results are more uniformly available, and these analyses can be pursued. To our knowledge, this is the largest structured review of basket trials in NDD conducted.

## Conclusions

Basket trials are an important tool in the armamentarium available to support drug development for NDDs. They are an efficient means of supporting proof-of-concept of an agent’s activity in two or more disorders sharing key biological or clinical features. Combined with biomarkers, they can be a powerful means of advancing precision drug development and precision medicine for patients with NDDs. With the advent of more informative tools to offer biological confirmation of the diagnosis of NDDs and monitor outcomes (e.g., positron emission tomography, fluid biomarkers), basket trials may be poised to be more useful in the quest to advance NDD treatments.

## Data Availability

The python and bash programming code, along with the data that supports the findings of this study, are available in Zenodo at 10.5281/zenodo.5717481 and in Github at https://github.com/SniperJF/NDDDrugMatcher/. Both links contain the same data for redundancy along with a description, or readme file, on how to reproduce the results. These data were derived from information available to the public at https://clinicaltrials.gov/ extracted on June 6, 2021.

## Abbreviations

AD: Alzheimer’s disease
ALS: Amyotrophic lateral sclerosis
CADRO: Common Alzheimer’s Disease Research Ontology
CBD: Corticobasal degeneration
CTE: Chronic traumatic encephalopathy
DLB: Dementia with Lewy bodies
DMT: Disease-modifying therapy
FDA: Food and Drug Administration
FTD: Frontotemporal dementia
FTLD: Frontotemporal lobal degeneration
HD: Huntington’s disease
LBD: Lewy body dementia
MOA: Mechanism of action
MSA: Multiple system atrophy
NIH: National Institutes of Health
NDD: Neurodegenerative disease
PD: Parkinson’s disease
PSP: Progressive supranuclear palsy
